# “BrainHeart”: Pilot Study on a Novel Application for Elderly Well-Being Based on Mindfulness Acceptance and Commitment Therapy

**DOI:** 10.3390/bioengineering11080787

**Published:** 2024-08-03

**Authors:** Roberta Bruschetta, Desiree Latella, Caterina Formica, Simona Campisi, Chiara Failla, Flavia Marino, Serena Iacono Isidoro, Fabio Mauro Giambò, Lilla Bonanno, Antonio Cerasa, Angelo Quartarone, Silvia Marino, Giovanni Pioggia, Rocco Salvatore Calabrò, Gennaro Tartarisco

**Affiliations:** 1 National Research Council of Italy, Institute for Biomedical Research and Innovation, C/O via Leanza, Istituto Marino, Mortelle, 98164 Messina, Italy; roberta.bruschetta@irib.cnr.it (R.B.); simona.campisi@irib.cnr.it (S.C.); chiara.failla@irib.cnr.it (C.F.); flavia.marino@irib.cnr.it (F.M.); serena.iaconoisidoro@irib.cnr.it (S.I.I.); giovanni.pioggia@cnr.it (G.P.); 2 IRCCS Centro Neurolesi “Bonino-Pulejo”, via Palermo S.S. 113—C.da Casazza, 98124 Messina, Italy; caterina.formica@irccsme.it (C.F.); fabio.giambo@irccsme.it (F.M.G.); lilla.bonanno@irccsme.it (L.B.); angelo.quartarone@irccsme.it (A.Q.); silvia.marino@irccsme.it (S.M.); roccos.calabro@irccsme.it (R.S.C.); 3 C.O.T. Cure Ortopediche Traumatologiche S.P.A, 98124 Messina, Italy; 4 Institute of BioImaging and Complex Biological Systems (IBSBC-CNR), via T. Campanella, 88100 Catanzaro, Italy; antonio.cerasa@cnr.it; 5 S. Anna Institute, 88900 Crotone, Italy

**Keywords:** mental health, mindfulness acceptance and commitment therapy (ACT), mobile application, well-being in seniors, usability

## Abstract

The rising prevalence of mental illness is straining global mental health systems, particularly affecting older adults who often face deteriorating physical health and decreased autonomy and quality of life. Early detection and targeted rehabilitation are crucial in mitigating these challenges. Mindfulness acceptance and commitment therapy (ACT) holds promise for enhancing motivation and well-being among the elderly, although delivering such psychological interventions is hindered by limited access to services, prompting exploration of remote delivery options like mobile applications. In this paper, we introduce the BrainHeart App (v.1.1.8), a mobile application tailored to improve physical and mental well-being in seniors. The app features a 10-day ACT program and other sections promoting healthy lifestyle. In a pilot study involving twenty participants, individuals engaged in daily mental exercises for 10 days using the app. Clinical evaluations, including assessments of psychological flexibility, overall cognitive profile, mindfulness disposition, cognitive fusion, and heart rate collected with Polar H10, were conducted at baseline (T0) and one month post-intervention (T1). Analysis revealed significant improvements in almost all neuropsychological scores, with high usability reported (system usability scale average score: 82.3 ± 9.31). Additionally, a negative correlation was found between usability and experiential avoidance (r = −0.51; *p* = 0.026), and a notable difference in heart rate was observed between baseline and post-intervention (F-value = 3.06; *p*-value = 0.09). These findings suggest that mindfulness-ACT exercises delivered via the BrainHeart App can enhance the well-being of elderly individuals, highlighting the potential of remote interventions in addressing mental health needs in this population.

## 1. Introduction

The number of people over 60 in the globe is predicted to reach two billion by 2050 [1]. Low- and middle-income countries (LMIC) will see an abrupt rise in the number of elderly citizens, which will have severe repercussions for these fragile economies [2]. Despite the common perception that older people are depressed, sluggish, and forgetful, many individuals live long, happy lives without experiencing any mental health issues. However, the number of people with mental illnesses is rising, which could have a detrimental effect on the world’s mental health systems as all nations’ mental health systems may soon reach capacity [3].

Over 20% of adults 55 years of age and older may be struggling with a mental health issue [3]. Mental illnesses can make medical conditions’ symptoms and functional limitations worse, as well as increase the need for medical resources, lengthen hospital stays, and raise overall costs of care [4]. The ability of an elderly person to perform fundamental daily tasks can be significantly impacted by mental health issues, which can lower their autonomy, independence, and quality of life [5].

As people age, their mental health is influenced by their physical and social surroundings as well as the accumulated effects of their past experiences and particular age-related stressors [3]. Psychological distress can be caused by a variety of factors, including considerable loss of intrinsic capacity, exposure to adversity, and a deterioration in functional ability [6]. The worry content of older adults generally tends to center around changes in health or possible developmental losses [7]. Adverse occurrences like bereavement, income declines, or a diminished feeling of purpose after retirement are more common among older persons [8].

Rehabilitative interventions aim to prevent further impairments and restore a person’s functional capabilities in this regard. Using adaptive techniques and resources, the main objective is to help patients carry out routine personal tasks without the help of a caregiver, or at the very least to eliminate the need for extra support [9]. Delaying the onset of frailty and preventing disability in the elderly hence depends on early and simple detection of the condition. This process can be achievable with the prompt application of strategic rehabilitation interventions. Recently, several aging and personal health care technologies have been proposed to help prevent, minimize, or alleviate some of the most frequent illnesses that afflict the elderly population [10].

Extensive research indicates that employing techniques based on cognitive behavioral therapy (CBT) can effectively enhance the motivation of elderly individuals to embrace a healthier lifestyle [11]. Notably, acceptance and commitment therapy (ACT) is establishing itself as a valuable support for promoting positive behavioral changes and well-being, especially in the elderly [12,13]. ACT, developed in the late 20th century by Steven C. Hayes, Kelly G. Wilson, and Kirk D. Strosahl, is a form of cognitive behavioral psychotherapy that employs experiential strategies involving acceptance and mindfulness, commitment to action, and behavior modification to enhance psychological flexibility. It offers a distinctive clinical perspective based on normal human language and cognition processes rather than specific cognitive or neurological deficits. ACT positively impacts mental health and well-being, and introduces coping strategies for stress reduction through mindfulness and acceptance techniques, preventing cardiovascular diseases [14]. Emphasizing values-based actions may encourage crucial behavioral changes, such as adopting healthier lifestyles [15].

A study by Jentsch and Wolf [16] examined the function of controlling negative emotions in enhancing psycho-physiological reactions to an acute psychosocial stressor. Difficulties in cognitive control and emotional regulation increase difficulty in coping with stressful factors and may be related to an altered autonomic balance [17].

An increased basal heart rate (HR) could be an autonomic response to distress [18] that reflects—with HRV—the dynamic relationship between sympathetic and parasympathetic nervous system influences [19]. An acceptance approach might play a role in flexible and adaptive responses to environmental demands, as well as increased somatic awareness and autonomic regulation, promoting a model of successful aging [7,20].

However, the effectiveness of prevention programs is restricted because the elderly experience emotional blunting by changes in social, body perception, and future expectations. 

Furthermore, the delivery of psychological interventions to older adults faces limitations stemming from poor access to available services, due to factors including physical limitations, limited awareness stigma, and financial constraints [21,22,23,24]. The utilization of remote delivery options for care emerges as a promising solution. Remote ACT-based approaches, employing interventions via telephone and utilizing work-at-home workbooks, have demonstrated effectiveness in addressing anxiety [25,26], depression [27,28], and comorbid conditions [29] among older adults. Therefore, healthcare services need to be innovative and respond to personalized requirements for improving the physical and mental health of the elderly population [30], who often must face long waiting times and difficulties accessing mental health care and who might not be able to afford frequent clinical visits.

In recent years, the pervasive integration of technology into our daily routines has given rise to mobile health (mHealth) solutions, such as mobile applications, designed to support both patients and healthcare professionals (HCPs) in disease management [31,32]. 

Mobile apps, as an alternative to traditional in-person methods, address numerous challenges in delivering therapy. They enable therapies at home, overcoming mobility issues, and offer flexibility to accommodate varying schedules, enhancing engagement. Additionally, they are cost-effective, include educational resources, facilitate progress monitoring, and allow content customization, collectively enhancing the overall therapeutic experience. Koh et al. [33] highlight both the potential benefits and inherent challenges of these digital tools in mental health interventions for the elderly. Firstly, mobile apps can overcome barriers of traditional healthcare by providing timely, cost-effective, and discreet support. This is particularly useful for older adults who may have difficulty traveling or feel embarrassed about seeking help. Most studies have focused on adolescents and young adults, leaving a gap in understanding how these factors impact older adults.

Based on these premises, in the following section, we present BrainHeart (BH) App, a mobile application designed to facilitate physical and mental well-being exercises for elderly individuals. Specifically, the primary aim of this study is to present a pilot investigation demonstrating the efficacy of integrating a 10-day ACT program into the app, alongside the utilization of other features, to promote a healthy lifestyle. The results of the first usability study are presented. 

## 2. Materials and Methods

### 2.1. BrainHeart App Overview

**Figure 1:**
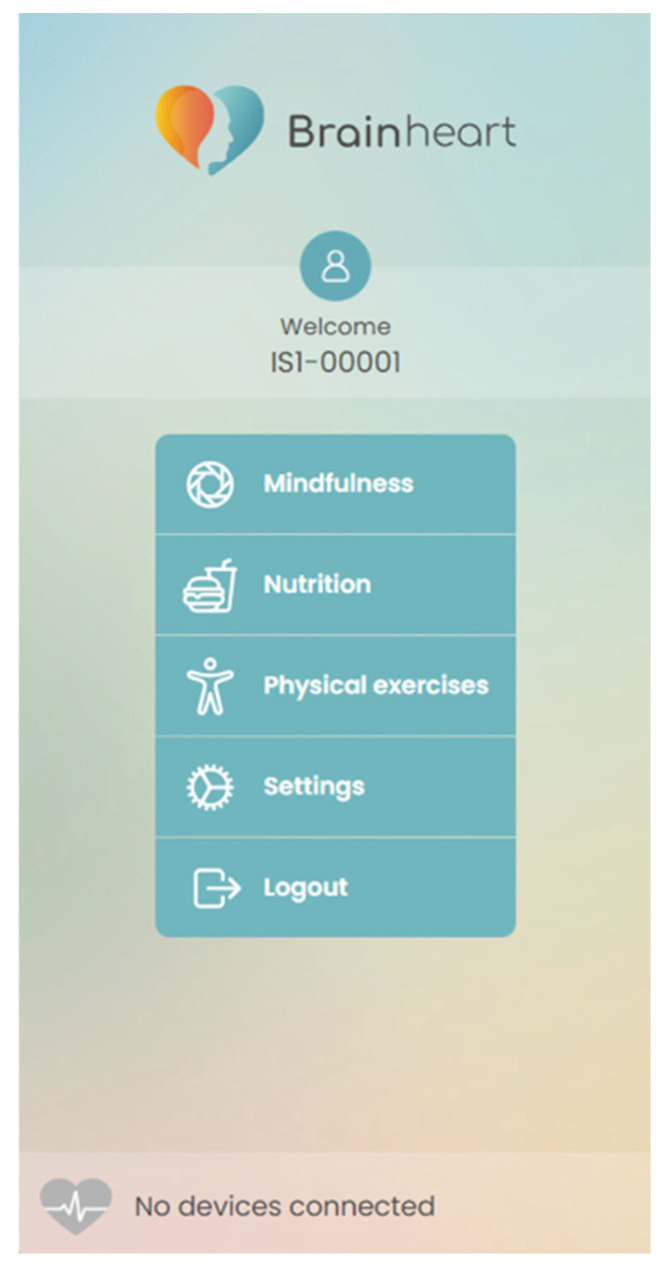
Sections of BrainHeart mobile application to promote overall psycho-physical well-being.

The BrainHeart App was developed within an Italian project (BRAINHEART—Sistemi intelligenti per la prevenzione delle malattie cardiovascolari. Project funded under the PO FESR Sicily 2014/2020—Azione 1.1.5.—n. 08749021035 http://progettobrainheart.com/) (accessed on 1 June 2022) aimed at developing new e-health systems for preventing heart disease. The developed e-health platform is a comprehensive system that integrates the monitoring of multiple parameters to enhance users’ lifestyles and, consequently, their overall psychophysical well-being. The platform includes dedicated sections for physical and mental exercises and nutrition advice, aiming to improve positive behavioral changes. The BH platform can be easily accessed through its user-friendly mobile application, which is available for free download on both the Apple Store and Google Play Store. Additionally, the platform provides a guided procedure for usage and navigation, ensuring an effective and efficient user experience. More precisely, the application integrates a structured protocol based on mindfulness-ACT cognitive behavioral therapy designed to support older people to understand the intricate relationship that exists between their feelings, thoughts, and the symptoms associated with their health condition. The intervention program targets their health-related concerns while simultaneously encouraging awareness and active listening, fostering stress management, and enhancing overall mental well-being through access to three specific dedicated sections (Figure 1). The platform also includes a web interface for caregivers and healthcare professionals to monitor users’ health status, allowing them to integrate and personalize mindfulness-ACT exercises, as well as physical activity and nutrition programs.

#### 2.1.1. Nutrition and Physical Exercises Sections

**Figure 2:**
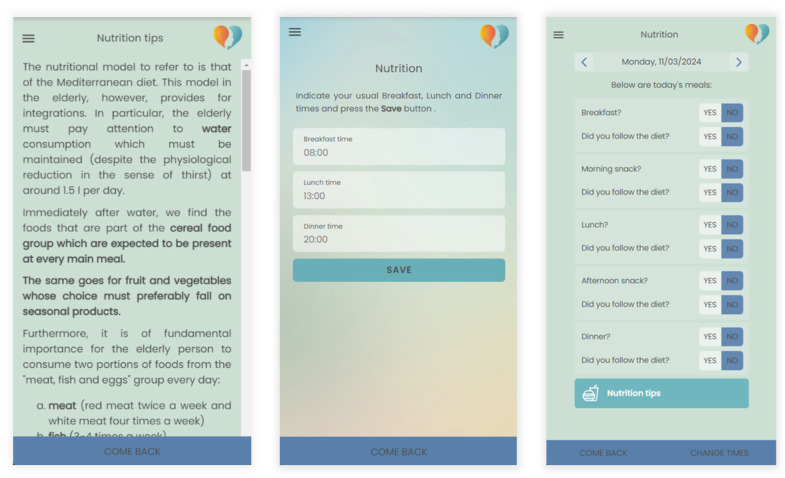
Example of nutrition tips and setting of dietary habits.

To enhance overall lifestyle, the platform actively encourages the adoption of healthy practices, such as maintaining a nutritious diet, engaging in regular exercise, staying well-hydrated, and ensuring sufficient sleep. To support participants in achieving these objectives, the app offers practical and realistic suggestions, recommending gradual and sustainable changes. For instance, the platform recommends dietary adjustments, incorporating light physical activities like walking or stretching, maintaining hydration throughout the day, and adhering to a proper sleep schedule. The BH platform includes dedicated sections for nutrition and physical activity. In the nutrition section, users gain access to fundamental nutritional information, enhancing awareness of their daily food choices. Additionally, users can log their mealtimes, promoting the regulation of dietary habits as reported in Figure 2. Through the platform, clinicians can deliver personalized nutritional advice to patients based on user-provided information, assisting in the improvement of dietary habits and the adoption of a more balanced diet. The platform evaluates users’ nutrition knowledge and behaviors, providing targeted suggestions to encourage mindful and healthy food choices.

**Figure 3:**
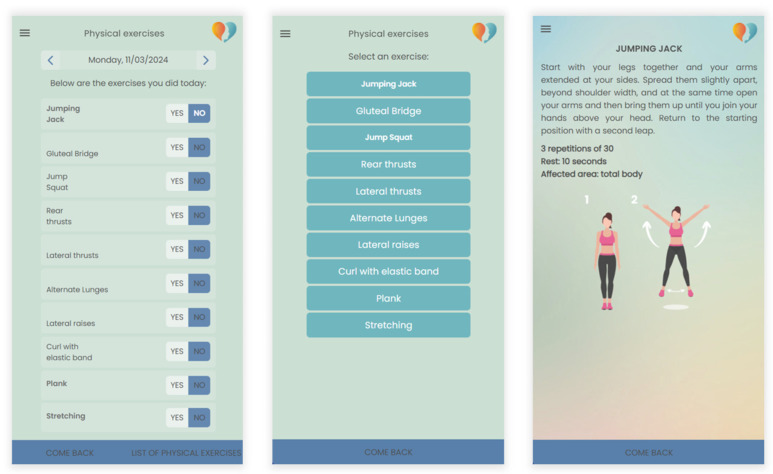
Example of personalized physical exercises program.

By monitoring physical activity, users can monitor their movement levels and receive real-time feedback, promoting an active and healthy lifestyle. The section also provides a comprehensive total body workout regimen, enabling the creation of personalized exercise programs based on individual needs and goals, emphasizing a tailored approach to fitness. The platform includes reminders for scheduled exercises with the flexibility to adjust appointments, ensuring the customization of physical activity (Figure 3).

**Figure 4:**
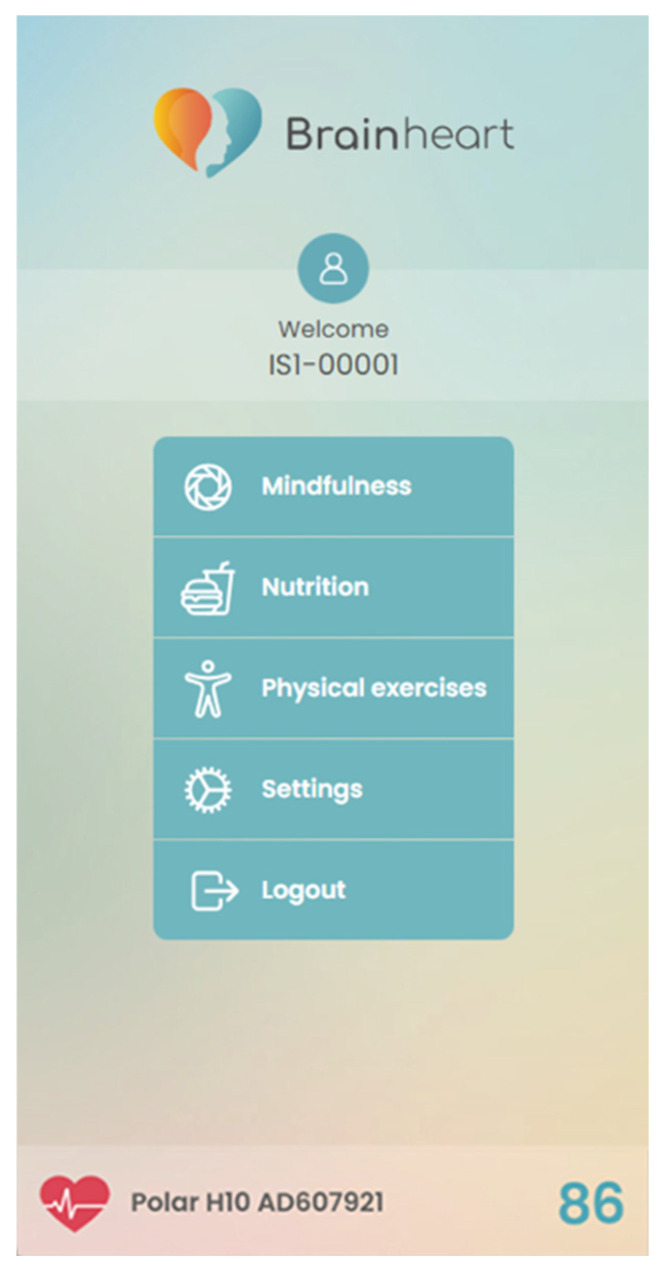
Example of data collection through the integration of a Polar H10 chest heat rate sensor.

The platform can be also integrated with wearable devices, enabling real-time data collection on physical activity, heart rate, and other metrics crucial for health monitoring and overall well-being. This integration was exemplified during our usability study, where the Polar H10 chest heart rate sensor has been integrated into comfortable every day-use T-shirts to facilitate its use. Through the sensor, the RR interval and corresponding heart rate variability were collected during each session to monitor the stress levels of the participants as shown in Figure 4.

#### 2.1.2. Mindfulness Section

The mindfulness exercises on the platform are designed to encourage a proactive mindset in older individuals regarding health prevention. Furthermore, the ACT exercises aim to enhance self-awareness and behavioral flexibility, encouraging the elderly to develop effective adaptation strategies. The goal is not merely to instruct meditation but to assist participants in expanding their awareness in daily life. This enables them to navigate diverse situations, live in the present moment, and effectively cope with the psychological aspects of their circumstances. The structure of the program includes a series of daily sessions, each focused on specific aspects of psychological flexibility, such as present moment awareness, acceptance of internal experiences, cognitive defusion, identification of personal values, and committed action. Each day, participants were invited to complete a guided mindfulness exercise via the app.

**Figure 5:**
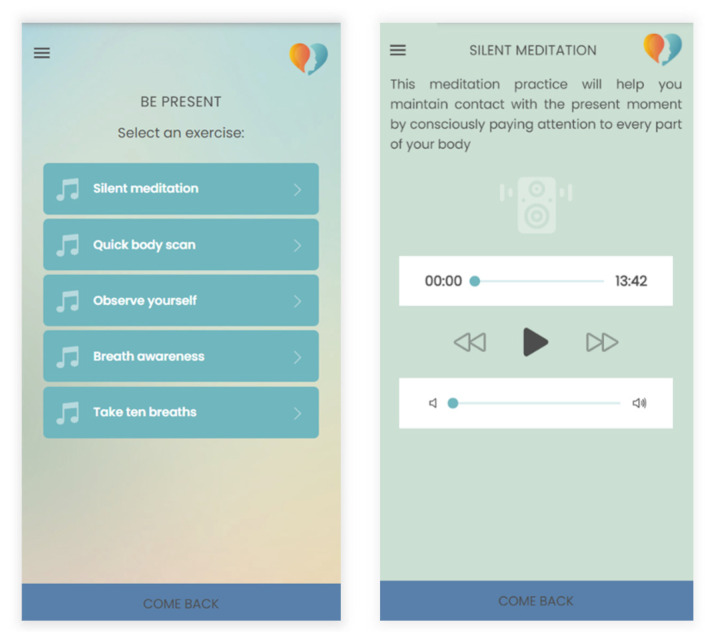
Example of meditation exercise included in the mindfulness section.

Meditation sessions were presented in a specific sequence to facilitate the progression from acquiring basic awareness skills to applying these skills in daily situations, focusing on two key aspects supporting individuals towards change: flexible awareness (being present) and openness (making space). By clicking on the “Being Present” button, users access a brief description of the contents. This description reports the section’s goal of assisting users in connecting with the present moment, directing their attention to the environment, and discerning what truly matters. Below this description, users encounter a “Continue” button, providing access to the next screen that lists five audio exercises (Figure 5). Instead, by clicking on the “Making Space” button, users are informed that the aim of this section is to help them make space and open to difficult thoughts and emotions by learning to choose not to struggle against them, shifting their attention to themselves and their actions. Clicking on “Continue” will open a list of four audio exercises related to the acceptance process. 

Mindfulness-ACT exercises could be summarized as follows:

1. Observing and letting go of thoughts, focusing on the present moment, with reflections on self-care and gratitude.
2. Heightened awareness of bodily sensations.
3. Emphasizes concentration on abdominal breathing and connecting with the present moment.
4. Emphasizes the normality of the mind wandering, observing breath, and refocusing attention.
5. Employing grounding techniques to connect with surroundings and manage thoughts and emotions.
6. Focusing on posture, breath, letting go of distractions, and emphasizing self-care and gratitude.
7. Focusing on breath and bodily sensations to deepen relaxation and awareness, encouraging gentle curiosity.
8. Encouraging awareness of bodily sensations, thoughts, and emotions, and connecting with the present moment.
9. Focusing on observing breath with curiosity, acknowledging distractions, and refocusing on breath.
10. Using all the senses to imagine a vivid scene to deepen relaxation and awareness, acknowledging distractions like thoughts or feelings, noticing them without judgment, and refocusing on the scene.

Many older adults have difficulty noticing and identifying internal experiences [7], so the program includes mindfulness exercises, such as mindful breathing, in order to develop and practice this skill. Participants are asked to reflect on what emotions they are experiencing, what physiological symptoms and bodily sensations emerge, and what they are thinking in the moment. After awareness of internal experiences is increased, exercises encourage the participant to increase acceptance of aversive internal events. Decreasing avoidance, the program works on being open to experience unpleasant emotions or thoughts without trying to modify or avoid them, demonstrating the futility of control. This process undermines the participant’s cognitive fusion with thought content, allowing them to achieve psychological distance from moment-to-moment experiences, promote psychological flexibility, and broaden the repertoire of behavioral and emotional responses.

The details of the meditation exercises are comprehensively listed in the Supplementary Table S1, including processes, exercise titles, brief descriptions, and durations.

### 2.2. Usability Tests

The usability evaluation is a fundamental step when developing new tools and technology that involve human–technology interaction. According to the international standard ISO 9241-11 [34], usability is defined as the extent to which specific users can effectively, efficiently, and satisfactorily achieve their goals using a product within a particular context. Effectiveness pertains to users’ abilities to achieve their goals, efficiency relates to the level of effort required by users, and satisfaction is users’ perceptions of the experience. These three key factors are influenced by user characteristics and goals, and the application’s context. Additionally, the standard outlines the necessary information to collect during usability analysis and provides guidelines for assessment. Tuena et al. [35] specifically emphasized the importance of assessing the usability of applications for elderly populations by identifying obstacles and facilitators, developing suitable tasks for the sample, defining usability criteria, and testing their clinical use. Within the context of the BH project, usability was evaluated using the system usability scale (SUS) [36,37], a widely used tool for assessing the usability of products, including web applications. The SUS comprises ten items presented in a questionnaire format. Participants rate their level of agreement on a Likert scale ranging from 0 to 4 for each statement. The final SUS score ranges from 0, indicating a lack of usability, to 100, denoting optimal usability and provides the overall satisfaction level of the sample. The SUS offers several advantages: it can be adapted to assess a wide array of technologies, it is quick and user-friendly for both users and researchers, it yields easily interpretable scores, and it has relatively low administration costs [37].

### 2.3. Participants

A total of 20 participants with ages between 50 and 85 (mean ± SD age: 65.4 ± 8.0 years; 50% males) were voluntary recruited between October and December 2022 at the I.R.C.C.S Centro Neurolesi “Bonino-Pulejo” in Messina, Italy. In order to minimize selection bias, the age range was set to between 50 and 85 years, reflecting the demographic of the clinic’s population. Exclusion criteria included: (i) dementia; (ii) moderate to severe depression; (iii) severe language disorders, including aphasia; (iv) other major psychiatric disorders; (v) praxis deficits. 

Specifically, as a screening instrument for detecting organic mental disorders or cognitive impairment, patients were administered the Mini-Mental State Examination (MMSE) [38] at baseline to assess their global cognitive profile. The MMSE is a brief, structured test of cognitive function widely used in clinical settings to evaluate cognitive abilities such as memory, attention, language, and visuospatial skills [38]. Participants were enrolled in the study with an MMSE score greater than 26, which generally indicates relatively intact cognitive function [39].

All subjects in the sample signed the informed consent and subsequently actively participated in a 10-day ACT cognitive behavioral therapy mobile app intervention, with a daily practice commitment of about 8–10 min. This study was approved by Ethics Committee Palermo I; Azienda Ospedaliera Universitaria (report nr. 04/2022—13 April 2022).

### 2.4. Clinical Assessment

Participants underwent evaluation at two distinct time points: initially during the recruitment, referred to as baseline (T0), and one month later (T1), post-intervention. Specifically, they were administered a series of self-report questionnaires aimed at investigating their psychological flexibility according to the Hexaflex Model [40].

The Hexaflex Model is a hexagonally shaped visual representation of the core processes and principles of ACT, illustrating how they interconnect to promote psychological flexibility. Psychological flexibility is the ability to remain open, adaptive, mindful, and effective in the presence of difficult or unwanted thoughts and emotions, preserving the capacity to act in alignment with one’s values and priorities.

The model is purposefully designed for the comprehensive classification and treatment of patients: each process within it represents a different aspect of psychological flexibility, providing therapists with a diverse array of activities and exercises to guide their patients during interventions.

**Figure 6:**
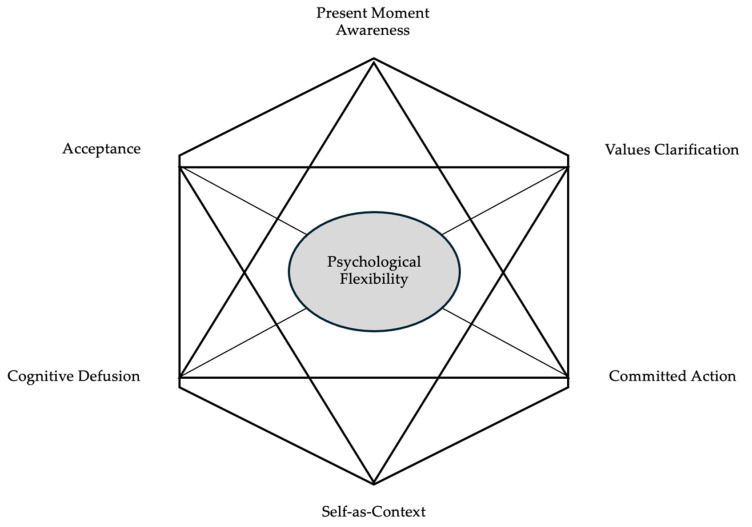
The Hexaflex Model.

Specifically, each of these processes—acceptance, cognitive defusion, self-as-context, present moment awareness, values, and committed action—has a dysfunctional counterpart, that upon identification requires attention and intervention: experiential avoidance, cognitive fusion, conceptualized self, dominance of the past and future, values restriction and confusion, and inactivity or impulsivity. The core of the hexagon-shaped ACT model, the Hexaflex (Figure 6), is psychological flexibility, the union of the six functional processes. 

Here in detail are the six core processes of the Hexaflex Model:

- Cognitive Defusion: Cognitive defusion involves creating distance from thoughts, reducing their impact. Instead of being fused with or dominated by thoughts, individuals learn techniques to observe thoughts without immediate attachment or identification. This allows for a more flexible and less reactive response to thoughts.
- Acceptance: Acceptance, in the ACT context, is about allowing thoughts and feelings to exist without struggling against them. Rather than trying to eliminate or control unwanted internal experiences, individuals practice accepting them as a natural part of the human experience. This process reduces the emotional struggle associated with difficult thoughts and emotions.
- Present Moment Awareness (Mindfulness): Mindfulness is the practice of being fully present in the current moment, paying attention to thoughts and feelings without judgment. It involves cultivating awareness and staying connected with the present rather than getting caught up in worries about the past or future.
- Self-as-Context: Self-as-context encourages individuals to recognize that they are not defined by their thoughts, feelings, or roles. Instead, they have a stable and consistent sense of self that remains unchanged by internal experiences. This process helps create a more resilient and less ego-identified sense of identity.
- Values Clarification: Values clarification involves exploring and clarifying the qualities and directions that a person values most in life. These values become a guide for decision-making and goal setting. Living in accordance with one’s values leads to a more meaningful and purposeful life.
- Committed Action: Once you have identified your values, committed action involves taking steps and making choices that align with those values. It is about moving in the direction of your values, even in the presence of difficulties or discomfort.

In addition to psychological flexibility, the overall cognitive profile, mindfulness disposition, and cognitive fusion of subjects were also assessed.

Specifically, the following self-report tests were employed:

- The Mindfulness Awareness Attention Scale (MAAS) [41], made up of 15 items, enables the assessment of a fundamental aspect of dispositional mindfulness: the open and receptive awareness of the present moment.
- The Cognitive Fusion Questionnaire (CFQ-13) [42], a 13-item questionnaire used to evaluate cognitive defusion—the capacity to create distance from one’s thoughts and memories, facilitating the pursuit of personal goals and values independent of internal events.
- The Multidimensional Psychological Flexibility Inventory (MPFI) [40], consisting of 60 items, to examine both psychological inflexibility and flexibility.

The battery of neuropsychological tests and the questionnaire investigating socio-demographic characteristics were included in a dedicated module for online administration through the application. Upon collection, data were automatically integrated into the platform, accessible for consultation by clinicians.

### 2.5. Protocol

**Figure 7:**
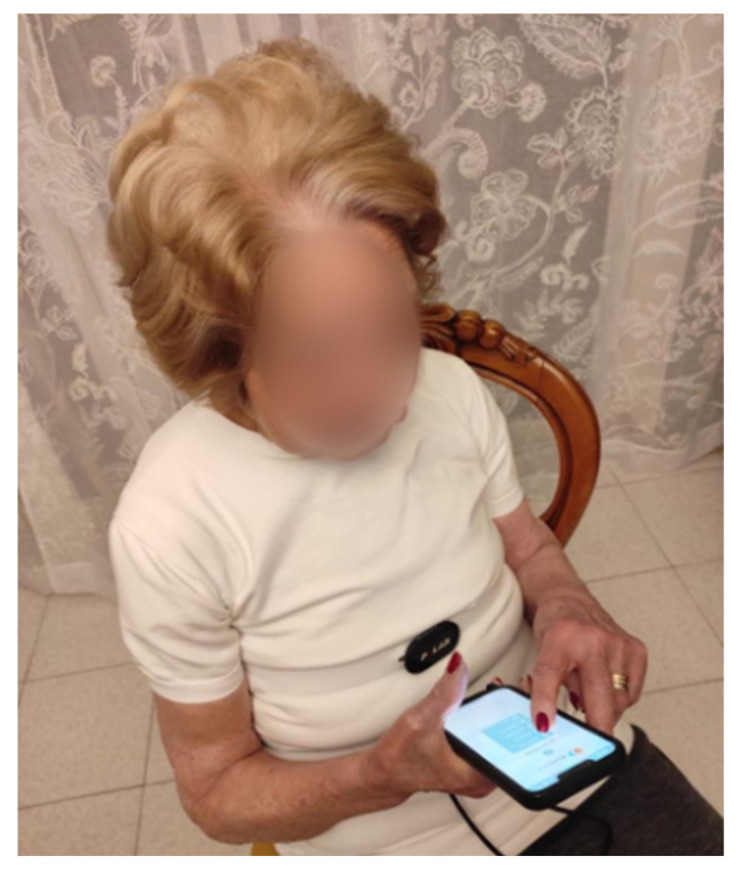
A participant wearing a T-shirt equipped with a Polar H10 sensor for measuring heart rate variability interacts with the BrainHeart application using her smartphone.

After the baseline assessment (T0), participants received detailed instructions to download and use the BH mobile app. Along with these instructions, they were provided with their login credentials and a wearable POLAR10 chest band integrated in a T-shirt for heart rate monitoring during the intervention protocol. Before each session, patients ensured the chest strap was properly moistened and adhered to the skin. After activating Bluetooth on their smartphones, they logged into the BH app using the provided credentials. The app established a Bluetooth connection with the heart rate sensor, recording the heart rate for 10 min (Figure 7). Subsequently, the strap was removed, and the patient initiated the mindfulness session within the app.

Each patient participated in a series of ten daily mindfulness sessions, with a random duration ranging from 1 min to 13 min. Access to these sessions was facilitated by the neuropsychologist, who unlocked them daily. Once an exercise was unlocked, participants had the option to repeat it at their discretion on the same day or in subsequent days. They were also encouraged to explore other sections of the app to assess its effectiveness in promoting a healthy lifestyle. The order of the mindfulness sessions was the same as that provided in the BrainHeart App Overview section. Adherence to the program was monitored through several methodologies integrated into the app. Firstly, the app automatically recorded the access and completion of each daily session by the participants, and the team had access to a dashboard that allowed for real-time monitoring of participants’ interactions with the app. This allowed us to track the number of sessions completed by each participant and to identify any usage patterns. Additionally, to promote adherence, the app included daily notifications reminding participants to complete the exercises. These notifications were scheduled to be sent at specific times of the day, chosen by the participants themselves, to better fit their daily routines. To collect qualitative data on user experience and improve the program, participants were asked to provide feedback through weekly questionnaires integrated into the app. These questionnaires included questions about the app’s usability, the quality of the mindfulness sessions, and the perceived impact of the exercises on their well-being. The responses were used to evaluate adherence and to make any necessary improvements to the program.

One month after the protocol (T1), participants received a notification to repeat the neuropsychological evaluation and were instructed to measure their heart rate for 10 min, following the same procedure as on previous days.

### 2.6. Statistical Analysis

For each participant, we computed the pre–post treatment variation (Delta T1-T0) for all the neuropsychological scores. Subsequently, we checked the distribution of these differences in each variable using the Shapiro–Wilk normality test. Given the non-normal distribution of differences across variables and the constraint of a small sample size (20 subjects), we chose to employ non-parametric methods for the subsequent analyses. To compare the variables between the two time points within the group, a paired Wilcoxon test was employed. Furthermore, upon calculating the changes in clinical scores between the baseline (T0) and one month later (T1), we conducted a correlation analysis between this variation and the system usability scale (SUS) using the Spearman method. We utilized analysis of covariance (ANCOVA) with repeated measures to examine the impact of the intervention on heart rate. Time points (T0 and T1) were considered as within-person factors, while age was included as a covariate in the model to account for its influence on changes in heart rate from T0 to T1. Using the same approach, we assessed whether the age had an impact on the variation in clinical scores between the two time points.

This statistical test was applied after verifying the assumptions using the test for homogeneity of variances (Levene), the test for normality (Shapiro–Wilk), and inspecting the residuals using the QQ plot. Analyses were performed using an open source R3.0 software package. A 95% confidence level was set with a 5% alpha error. Statistical significance was set at *p* < 0.05.

## 3. Results

**Figure 8:**
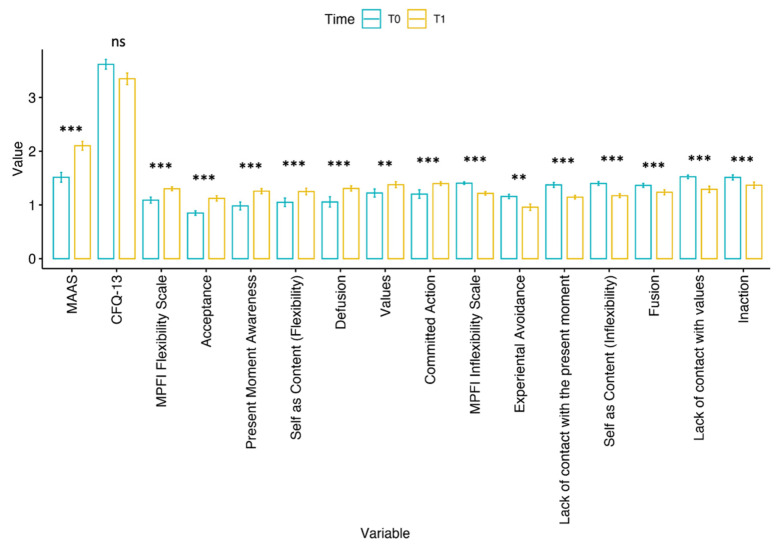
Bar plot of the assessed scores at T0 and T1 with significance level from paired-samples Wilcoxon test. Legend: ns = *p* > 0.05, ** = *p* ≤ 0.01, *** = *p* ≤ 0.001. Prior to visualization, all scores were normalized through logarithmic transformation.

Considering neuropsychological evaluation before and after treatment, the analysis revealed significant differences within the group when comparing T0 and T1 for all scores, except for the total CFQ-13 (Figure 8). Details about the paired-samples Wilcoxon statistical test results are reported in Table 1. 

**Figure 9:**
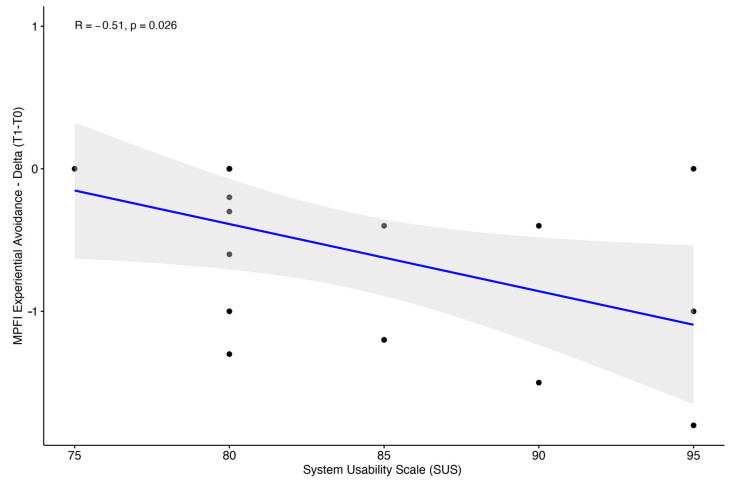
Scatter plot between system usability scale and experiential avoidance variation (T1-T0) with Spearman correlation coefficient and *p*-value.

Analysis of usability revealed an SUS average score of 82.3 (±9.31), with a range from 52 to 95. Moreover, we identified a significant negative correlation between SUS and experiential avoidance (r = −0.51; *p* = 0.026) (Figure 9). 

Finally, evaluating the heart rate changes before and after treatment, the ANCOVA analysis demonstrated a notable difference in heart rate between T0 and T1, considering the effect of age as a covariate (F-value = 3.06; *p*-value = 0.09). Descriptive statistics of heart rate grouped by time points are reported in Table 2.

## 4. Discussion

In this study, we introduced a novel mobile application designed to support the psycho-physical well-being of frail individuals, employing principles of CBT and ACT. These established psychotherapeutic approaches aim to enhance psychological flexibility, emotional regulation, and cognitive restructuring. The mindfulness-ACT exercises integrated into our application combine mindfulness meditation with aspects of cognitive therapy to address rumination and negative thought patterns. Moreover, ACT focuses on the acceptance of thoughts and emotions while encouraging behavior aligned with personal values. Both CBT and ACT have demonstrated effectiveness in alleviating stress, anxiety, and depressive symptoms. 

By employing these approaches, our application aims to provide a tool for improving mental health and overall well-being in vulnerable populations.

Preliminary examination revealed evidence of a notably high level of usability, with a sample size of 20 participants considered robust for an initial evaluation. Research supports that even with relatively small sample sizes, meaningful insights into usability can be obtained. For instance, in [43], Nielsen argues that testing with five participants can uncover up to 85% of usability issues, while larger samples provide additional confidence and context for these findings. Thus, the inclusion of 20 subjects provides a solid foundation for assessing usability, offering a preliminary yet significant understanding of the platform’s effectiveness.

Additionally, the observed negative correlation between the SUS score and the experiential avoidance MPFI subscale suggests that individuals with lower SUS scores may exhibit higher levels of avoidant coping styles. This finding indicates that those who experience lower usability scores might be more prone to avoidant coping strategies, which can include cognitive methods such as rumination or preoccupation, and behavioral strategies like withdrawal or procrastination. These avoidant behaviors have been associated with increased levels of distress and diminished psychological well-being [44]. Consequently, users who engage in these avoidance strategies may find the platform more challenging to use and less satisfying. Further research is necessary to confirm these hypotheses and explore their implications for user experience and design.

Furthermore, data collected and analyzed in our pilot study demonstrated significant improvements in most clinical scores within the experimental group. This suggests the effectiveness of delivering mindfulness-ACT exercises through the BH program to support vulnerable individuals, allowing them to better handle stress and everyday challenges.

The significant differences observed in the MAAS and MPFI scores reflect an increase in mindfulness awareness and an improved capacity to fully engage with and accept unwanted experiences. This also indicates a heightened ability to remain connected with one’s experiences while maintaining a broader perspective of the self. Additionally, the results suggest an enhanced skill in distancing oneself from unwanted experiences without becoming trapped by them, thereby supporting behavior aligned with important life goals. Changes in the MPFI Inflexibility Scale further demonstrate an improved ability to attend to one’s present experiences without judgment, which prevents a narrowed self-view and promotes consistent behavior in line with personal values and meaningful aspects of life.

Several literature studies have shown that mindfulness techniques associated with cognitive behavioral techniques are effective for the treatment of a wide range of physical and behavioral disorders in the face-to-face modality [17,20,25,29]. An acceptance approach in which individuals learn to focus on their remaining resources seems to be more beneficial than an approach in which they are encouraged to modify their thinking; accepting declines that are unchangeable followed by identifying goals that are still attainable is adaptive and beneficial [7].

Integrating ACT into the BH app offers a set of mechanisms that can enhance users’ digital well-being. By developing psychological flexibility, promoting present moment awareness, encouraging acceptance, clarifying personal values, and providing physiological support and accessibility, the app can significantly contribute to users’ mental and physical health. Conditions that could benefit from integrating this mindfulness-ACT-based approach into the app include cardiovascular diseases, which are currently the leading cause of death worldwide. Extensively documented in the literature, these diseases are closely linked to lifestyle factors and the development of psychological disorders such as depression [45]. 

To our knowledge, this study is the first to examine the usability of an app-based mindfulness-ACT program with nonclinical, community-dwelling elderly adults, demonstrating a decrease in basal heart rate in just 10 days. Other studies have shown potential benefits for well-being in 14- [46] or 30-day [47] interventions with older adults, without any reference to physiological parameters. The integration of physiological monitoring, such as heart rate, allows users to receive real-time feedback on their physical state. This monitoring, combined with mindfulness and ACT exercises, helps users develop greater body awareness and better regulate stress responses. Although the *p*-value of 0.09 does not meet the conventional threshold for statistical significance, it suggests a potential trend indicating that mindfulness-ACT exercises may play a role in fostering flexible and adaptive responses to environmental demands and reducing potential cardiovascular risk. Further exploration in future studies with larger sample sizes is warranted.

In addition, using a mobile app to implement an ACT protocol presents numerous benefits. It ensures accessibility and flexibility, enabling users to seamlessly incorporate therapy into their daily routines regardless of their location. The app’s regular support and reminders help users stay committed to their therapeutic practices. Moreover, mobile apps can deliver personalized exercises and strategies tailored to each individual’s progress and responses, while also providing immediate feedback and tracking effectiveness through data collection. They offer a level of privacy and anonymity that can make addressing sensitive issues more comfortable [33]. Additionally, apps are typically more cost-effective than traditional therapy sessions, broadening access to ACT. Finally, by supporting ongoing practice beyond formal treatment, apps contribute to sustained well-being over the long term. Our results highlighted that the BH app is effective in motivating patients to change their lifestyle and adopt healthier behaviors to improve their quality of life. Mobile mental health apps hold unique potential to supplement traditional interventions by providing accessible and stigma-free support, but it is essential to manage associated risks such as engagement challenges. The elderly in fact might face greater challenges in using technology compared to younger generations. Therefore, it is crucial that mental health apps are designed to be effective and user-friendly for older adults [33]. Our study focused on the functionality of the app and the satisfaction associated with its use to contribute to the full realization of the benefits of this digital tool for the elderly population.

However, as a pilot study, this research has some limitations. The constrained duration of the treatment poses challenges in accurately estimating long-term outcomes. Short-term studies may not capture the sustainability of benefits or potential delayed effects, which are crucial for assessing the true effectiveness of the intervention. Additionally, the absence of a control group limits our ability to attribute observed changes specifically to the mindfulness-ACT intervention, as opposed to other external factors or placebo effects. Nonetheless, the primary aim of this pilot study was to preliminarily explore the potential benefits associated with the BH app. Furthermore, we are currently planning additional studies, including comparisons with control groups, in the upcoming RESILIENT project (details provided in the Funding Section) to provide a more comprehensive understanding of the intervention’s impact.

## 5. Conclusions

Based on our preliminary findings, the “BrainHeart” app shows promise as a commercial tool that can enhance overall well-being and reduce the risk of various health conditions by providing an effective, accessible, and user-friendly mobile app. The app primarily aims to improve quality of life and self-management in patients by integrating several intervention modalities in the domains of physical health management and psychological support, especially for the elderly. This perspective supports the idea that patient empowerment is a key objective, as it emphasizes that patients should not rely solely on caregiver supervision (which may not always be available) and should make healthy lifestyle choices independently. The results suggest that mindfulness-ACT exercises may promote flexible and adaptive responses to environmental demands and have potential in reducing cardiovascular risk, thus potentially improving overall quality of life. For existing fields of study, this research offers a novel approach to digital mental health interventions and highlights the need for future clinical trials to compare its efficacy with traditional treatments, paving the way for further advancements in mobile health technologies. However, it is essential to conduct future clinical trials with a larger and more diverse population, including individuals with frailty and common health issues such as depression, anxiety, or cardiovascular disease. These trials will allow us to compare the efficacy of our mobile application with traditional treatments and provide a more comprehensive assessment of its potential as an innovative technological solution. Additionally, we plan to develop further exercises targeting various cognitive and emotional domains to enhance the app’s effectiveness.

## Figures and Tables

**Table 1:** Results of paired-samples Wilcoxon test between T0 and T1.

Variable	Statistics	*p*-Value	Mean Difference
MAAS	0.0	<0.001	−3.500
CFQ-13	99.0	0.297	5.000
MPFI Flexibility Scale	0.0	<0.001	−0.700
Acceptance	0.0	<0.001	−0.900
Present Moment Awareness	0.0	<0.001	−1.050
Self as Content (Flexibility)	0.0	0.001	−0.800
Defusion	0.0	0.001	−0.950
Values	0.0	0.004	−0.950
Committed Action	0.0	0.001	−0.500
MPFI Inflexibility Scale	210.0	<0.001	0.675
Experiential Avoidance	78.0	0.003	0.900
Lack of contact with the present moment	136.0	<0.001	1.000
Self as Content (Inflexibility)	153.0	<0.001	0.950
Fusion	131.5	0.001	0.500
Lack of contact with values	171.0	<0.001	0.900
Inaction	152.0	<0.001	0.650

**Table 2:** Descriptive statistics of heart rate grouped by time point.

Time Point	Mean	Median	SD	Range	Minimum	Maximum
T0	81.5	80.4	9.60	34.1	66.6	100.7
T1	75.3	75.5	10.95	47.9	46.0	94.0

## Data Availability

Dataset available on request from the corresponding authors.

## Supplementary Materials

The following supporting information can be downloaded at: https://www.mdpi.com/article/10.3390/bioengineering11080787/s1, Table S1: Details of the meditation exercises included in the mindfulness section.
