# Supplementary material for: “BrainHeart”: Pilot Study on a Novel Application for Elderly Well-Being Based on Mindfulness Acceptance and Commitment Therapy
Source: Bioengineering (Basel). 2024 Aug 3;11(8):787. doi: 10.3390/bioengineering11080787 (PMC11351599; doi:10.3390/bioengineering11080787)
Supplement: Supplementary file 1 [file bioengineering-11-00787-s001.zip › bioengineering-3112390-supplementary.pdf]

Table S1. Details of the meditation exercises included in the mindfulness section.

N	Process	Name	App Description	Summary of the exercise audio	Duration
1	Being present	Silent Meditation	"This meditative practice will help you stay in touch with the present moment by mindfully paying attention to each part of the body."	The audio guides the listener through a meditation practice. It begins with instructions on sitting comfortably, focusing on posture, and letting go of distractions. The practice involves observing sounds and sensations with kindness and curiosity, at first that relate to hearing and then shifting attention to the breath and sensations in the body. The practice continues with observing and letting go of thoughts, bringing attention back to the present moment. The meditation concludes with reflections on the practice, emphasizing self-care and gratitude.	9:25
2	Being present	Quick body scan	"The body scan is a guided meditation designed to help you focus on the sensations in your body. It's a kind of guided tour through the sensations of the body, inviting you to cultivate openness and curiosity rather than being carried away by judgments and stories."	The audio leads a meditation practice, prompting the listener to close their eyes, focus on breath, and attend to bodily sensations, especially points of contact with the chair or floor. Emphasis is on letting go with each exhale, deepening relaxation. The aim is heightened awareness, not altering feelings, directing attention to each body part. The meditation explores sensations from abdomen to toes, utilizing breath and visualization. It encourages gentle curiosity, acknowledging distractions, and redirecting focus to the body. Systematically guiding attention, the audio prompts observation of sensations and breath awareness. It concludes with minutes of overall body	13:38

N	Process	Name	App Description	Summary of the exercise audio	Duration
				awareness, returning gently to the present surroundings.	
3	Being present	Observing oneself	"The focus of this exercise is centered on developing a sense of Self as the observer of your experiences moment by moment."	The text guides the reader through a self-reflection exercise. It encourages asking the question, "What is happening inside me right now?" and becoming aware of bodily sensations in the current environment. It prompts observation of the state of the mind, identifying emerging thoughts and current emotions. The focus then shifts to breath, emphasizing concentration on abdominal breathing and gently redirecting attention back if thoughts wander. The listener is instructed to be aware of sensations from feet to head, treating the body as a single breathing entity. Facial expression and connection with all senses are highlighted, urging an awareness of the surrounding world's shapes and colors. The overall message is to connect with the present moment, ready to act or do nothing, as preferred.	1:27
4	Being present	Breath awareness	"This exercise will help you observe the body in relation to inhalation and exhalation, keeping you connected to the present moment."	This guided exercise invites you to sit with your feet firmly on the ground and your back straight, either fixing your gaze on a point or closing your eyes. The focus is on observing your breath as if you were a curious scientist encountering it for the first time. The narration guides you through various aspects of your breath, inviting you to notice the air entering and leaving through your nostrils, the subtle movements of your shoulders,	6:56

N	Process	Name	App Description	Summary of the exercise audio	Duration
				chest, and abdomen. Throughout the exercise, distractions, whether emotional or cognitive, are acknowledged with gentleness, and the instruction is to continuously return your attention to your breath. The narrative emphasizes the normality of the mind wandering and provides reassurance that, each time it happens, the goal is simply to recognize the distraction, refocus on the breath, and continue the practice. The exercise concludes by bringing awareness back to the present moment in the room and opening your eyes.	
5	Being present	Take ten breaths	"This is a simple exercise to center yourself and connect with your environment. Practice it throughout the day, especially whenever you find yourself caught in your thoughts and emotions."	This exercise helps you center yourself and connect with your surroundings. Take ten slow breaths, observing the sensations in your lungs and body. Allow thoughts to come and go like passing cars. Notice your breath, body, and surroundings. In the "Grounding Anchor" exercise, plant your feet, feel the floor's support, and be aware of your body. Look around, noting what you see and hear. Practice when caught in thoughts and emotions.	3:23
6	Making space	Steps of self-compassion	"This meditative practice will help you stay in touch with the present moment by mindfully paying attention to each part of the body."	The audio guides the listener through meditation practice. It begins with instructions on sitting comfortably, focusing on posture, and letting go of distractions. The practice involves observing sounds and sensations with kindness and curiosity, at first that relate to hearing and then shifting	4:40

N	Process	Name	App Description	Summary of the exercise audio	Duration
				attention to breath and sensations in the body. The practice continues with observing and letting go of thoughts, bringing attention back to the present moment. The meditation concludes with reflections on the practice, emphasizing self-care and gratitude.	
7	Making space	Notice your emotions	“The body scan is a guided meditation designed to help you focus on the sensations in your body. It's a kind of guided tour through the sensations of the body, inviting you to cultivate openness and curiosity rather than being carried away by judgments and stories.”	The audio leads to meditation practice, prompting the listener to close their eyes, focus on breath, and attend to bodily sensations, especially points of contact with the chair or floor. Emphasis is on letting go with each exhale, deepening relaxation. The aim is heightened awareness, not altering feelings, directing attention to each body part. The meditation explores sensations from abdomen to toes, utilizing breath and visualization. It encourages gentle curiosity, acknowledging distractions, and redirecting focus to the body. Systematically guiding attention, the audio prompts observation of sensations and breath awareness. It concludes with minutes of overall body awareness, returning gently to the present surroundings.	8:06
8	Making space	Passengers on the bus	"The focus of this exercise is centered on developing a sense of Self as the observer of your experiences moment by moment."	The text guides the reader through a self-reflection exercise. It encourages asking the question, "What is happening inside me right now?" and becoming aware of bodily sensations in the current environment. It prompts observation of the state of the mind, identifying	4:18

N	Process	Name	App Description	Summary of the exercise audio	Duration
				emerging thoughts and current emotions. The focus then shifts to breath, emphasizing concentration on abdominal breathing and gently redirecting attention back if thoughts wander. The listener is instructed to be aware of sensations from feet to head, treating the body as a single breathing entity. Facial expression and connection with all senses are highlighted, urging an awareness of the surrounding world's shapes and colors. The overall message is to connect with the present moment, ready to act or do nothing, as preferred.	
9	Making space	You are like the sky and the clouds	"This exercise will help you observe the body in relation to inhalation and exhalation, keeping you connected to the present moment."	This guided exercise invites you to sit with your feet firmly on the ground and your back straight, either fixing your gaze on a point or closing your eyes. The focus is on observing your breath as if you were a curious scientist encountering it for the first time. The narration guides you through various aspects of your breath, inviting you to notice the air entering and leaving through your nostrils, the subtle movements of your shoulders, chest, and abdomen. Throughout the exercise, distractions, whether emotional or cognitive, are acknowledged with gentleness, and the instruction is to continuously return your attention to your breath. The narrative emphasizes the normality of the mind wandering and provides reassurance that, each time it happens, the goal is simply to recognize the distraction,	1:37

N	Process	Name	App Description	Summary of the exercise audio	Duration
				refocus on breath, and continue the practice. The exercise concludes by bringing awareness back to the present moment in the room and opening your eyes.	
10	Making space	Leaves on a stream	"This exercise will help you gain perspective on your thoughts and feelings, reducing their power."	Imagine you are resting by the side of a stream. This scene can look however you like. Use all your senses to imagine what the stream and its surroundings look like, the sound of the water and other ambiance, the physical sensations, and anything else that comes to mind.<br>The focus is on simply observing the stream. When distractions enter your mind, such as thoughts or feelings, take a moment to notice them without judgment.<br>After taking a moment to observe a thought or feeling, return your attention to the stream. Place your thought upon a leaf.<br>Place the leaf in the water and watch it float down the stream until it disappears.<br>Continue visualizing the stream, simply observing and releasing any thoughts that enter your mind.	10:33
